# Error in Conflicts of Interest Section

**DOI:** 10.1001/jamahealthforum.2024.2859

**Published:** 2024-08-23

The Viewpoint titled “Artificial Intelligence Can Be Regulated Using Current Patient Safety Procedures and Infrastructure in Hospitals,”^1^ published on June 28, 2024, was corrected to fix an error in the Conflicts of Interest section. Specifically, where it previously read “Dr Economou-Zavlanos reported volunteer and paid work for the Center for Human-Compatible Artificial Intelligence (CHAI); has co-led operations since its foundation and was recently appointed as CHAI’s Scientific Director; is a member of the Trustworthy and Responsible AI Network (TRAIN),” it now reads “Dr Economou-Zavlanos reported volunteer and paid work for the Coalition for Health AI (CHAI); has co-led operations since its foundation and was recently appointed as CHAI’s Scientific Director; is a member of the Trustworthy and Responsible AI Network (TRAIN).”
